# Supplementary material for: COVID-19 infodemic on Facebook and containment measures in Italy, United Kingdom and New Zealand
Source: PLoS One. 2022 May 19;17(5):e0267022. doi: 10.1371/journal.pone.0267022 (PMC9119508; doi:10.1371/journal.pone.0267022)
Supplement: S1 File — (PDF) [file pone.0267022.s001.pdf]

COVID-19 infodemic on Facebook and containment measures in Italy, United Kingdom and New Zealand

Gabriele Etta[2], Alessandro Galeazzi[3], Jamie Ray Hutchings[1], Connor Stirling James Smith[1], Mauro Conti[4], Walter Quattrociocchi[2], Giulio Valentino Dalla Riva[1],

1 School of Mathematics and Statistics, University of Canterbury, New Zealand

2 Center of Data Science and Complexity for Society, Department of Computer Science, Sapienza Università di Roma

3 Department of Information Engineering, University of Brescia

4 Department of Mathematics, University of Padova, Italy

Supporting Information

1 Data Collection and Filtering Procedure

Data can be obtained by using Crowdtangle [1], requesting access to their Crowdtangle Academical Program [2]. A Facebook dashboard is required to obtain the posts used in the study. Furthermore, a search operation from 1/1/2020 to 31/12/2020 must be done by looking for pages whose admin is located in one of the three countries in the study and groups. The lists of keywords employed in the search for each country are listed in the *italian_keywords.txt* and *english_keywords.txt* files respectively, available at the online repository. Both files can be found at the repository of the paper [3].

After having collected posts from Crowdtangle, a filter operation must be done by retaining only those posts with a link to a news outlet. The list of news outlets employed for this study can be found on Media Bias / Fact Check [4]. After that, a set of comparable results can be obtained by following the methodology expressed in the paper.

[1]https://help.crowdtangle.com
[2]https://help.crowdtangle.com/en/articles/4302208-crowdtangle-for-academics-and-researchers
[3]https://osf.io/vmezh/
[4]https://mediabiasfactcheck.com/

We do not have any exclusive access to datasets. As previously expressed, Crowdtangle research programs can be joined by all the academics and researchers interested in this kind of analysis upon a reasonable request.
