# Supplementary material for: COVID-19 infodemic on Facebook and containment measures in Italy, United Kingdom and New Zealand
Source: PLoS One. 2022 May 19;17(5):e0267022. doi: 10.1371/journal.pone.0267022 (PMC9119508; doi:10.1371/journal.pone.0267022)
Supplement: Attachment — Submitted filename: Response to Reviewers.pdf [file pone.0267022.s002.pdf]

Prof. Walter Quattrociocchi
Sapienza University of Rome
Piazzale Aldo Moro 5, 00185 Roma
walter.quattrociocchi@uniroma1.it

Dear Editor,
Thank you very much for handling our manuscript and for obtaining reviews for it. We are delighted to receive such a response. We are happy that the Referees consider our work positively. We are also flattered to accept the Editor's congratulations.

As requested, we carefully revised our paper. We addressed all the points raised by the reviewers and provided more details on the research context. Please find below the point-to-point answers to Referees' comments. We thank the referees for their accurate perusal of our work and the encouraging words on our research's relevance.

Reviewer #1: Paper is clearly written and lays out methodology and findings clearly and concisely. The association between the infodemic and other factors (SI, case count) are clear but recommend including more detail in the discussion on how these interactions affect the health and health behaviors of community members.

We thank the reviewer for the comment. We further extended the discussion by describing the role that the association has in the citizens' perspective, calling for actions from policy makers. Therefore, we added the following part at Page 8, Section 3: "*Indeed, the existence of a role for the number of cases, the news media coverage, and the strictness of containment measures in the evolution of infodemic is a pivotal point for policy-makers to take decisions for the safety of community members. Such decisions should consider that effective communication strategies must be developed and followed when publishing and circulating news, knowing that people, especially during a severe epidemic spreading, may be more susceptible to the content proposed. Furthermore, further actions are required to increase citizen awareness and understanding of the pandemic.*"

Reviewer #2: This study uses a novel approach to examine the relationship between the information environment of a specific social media platform and broader COVID-19 epidemiological/social developments. The study findings have important implications for understanding the dynamics of infodemics in the context of other public health data, including epidemiological data. However, there are some important methodological limitations/questions that warrant further discussion, which are listed

below. Some of these limitations ideally should be addressed in the discussion section.

1.) The list of keywords used to identify Facebook posts appears to be narrow. For example, it does not have slangs or other indigenous categories likely to be used by disinformation actors or misinformed consumers. The dataset may therefore be inherently biased towards more "credible" posts.

We thank the reviewer for the comment. We address the limitations that this keyword selection may cause due to the lack of slangs or other indigenous categories. We tried to be as comprehensive as possible by searching for terms with a common root but which could diversify in their postfix. However, there exists several papers that used a similar techniques to gather data, without considering slang/indigenous categories, see for example the work of:

- Yang, Kai-Cheng, et al. "The COVID-19 infodemic: twitter versus facebook." Big Data & Society 8.1 (2021): 20539517211013861 and Kiran Garimella, Gianmarco De Francisci Morales, Aristides Gionis
- Michael Mathioudakis. 2018. Political Discourse on Social Media: Echo Chambers, Gatekeepers, and the Price of Bipartisanship. In Proceedings of the 2018 World Wide Web Conference (WWW '18).

2.) Binary categorization of "questionable" vs. "reliable" Facebook posts seems somewhat limiting. A post can have a link to a credible source and still interpret it in a misleading way, or people can spam the comment section with misleading claims even if the original post only presents credible information.

We thank the reviewer for the comment. The misleading interpretation of posts is a phenomenon whose measurement is hard to be performed. Similarly, the spamming of misleading claims in the comment section of credible posts cannot be measured since CrowdTangle does not provide the comments of posts. Therefore, the binary categorization of posts into “Questionable” and “Reliable” has the only goal to understand the resonance of news outlets who are recognized as conspiracists or promoters of pseudoscience compared with more factual ones. In the end, we summarized these observations in the

paragraph concerning the limitations of the study. Indeed, in Section 3, page 8, we added the following part "*First, as CrowdTangle does not provide the content of the comments, we can not investigate whether people are inclined to spam the comment section with misleading claims even if the original post only presents credible information". Coherently, we changed "For Example" at line 180 with "Furthermore".*

For the sake of completeness, we report to the reviewer a list of influential works that perform the same kind of categorization, employing MBFC:

1) Cinelli, M., Quattrociocchi, W., Galeazzi, A. et al. The COVID-19 social media infodemic. Sci Rep 10.

2) Shao, C., Ciampaglia, G.L., Varol, O. et al. The spread of low-credibility content by social bots. Nat Commun 9, 4787 (2018).

3) Del Vicario, Michela, et al. "The spreading of misinformation online." Proceedings of the National Academy of Sciences 113.3 (2016): 554-559.

3.) The fact that the co-authors only focus on posts with a link significantly reduces the amount of data analyzed - this is a bit concerning especially because CrowdTangle is already limiting in the sense that it only picks up a subset of all posts available on Facebook i.e., public content from verified accounts.

We thank the reviewer for the comment. We added the reviewer's observation as a potential limitation of the study explained in Section 3, Page 8, with the following part "First, as CrowdTangle does not provide the content of the comments, we can not investigate whether people are inclined to spam the comment section with misleading claims even if the original post only presents credible information. *Second, the study focuses only on those posts with a link* to perform the categorization of news outlets with MBFC. *We are aware that this methodology reduces the study sample, which CrowdTangle already restricts since it provides only posts from public Facebook pages with more*

than 25K Page Likes or Followers, public Facebook groups with at least 95K members, all US-based public groups with at least 2K members and all verified profiles.”. Furthermore, we provide a list of papers which categorized posts through the same rationale:

1) Bovet, Alexandre, and Hernán A. Makse. "Influence of fake news in Twitter during the 2016 US presidential election." Nature communications 10.1 (2019): 1-14.
2) Kiran Garimella, Gianmarco De Francisci Morales, Aristides Gionis, and Michael Mathioudakis. 2018. Political Discourse on Social Media: Echo Chambers, Gatekeepers, and the Price of Bipartisanship. In Proceedings of the 2018 World Wide Web Conference (WWW '18).

4.) The MBFC scale used to classify posts appears to be based on the US political landscape according to their website. It's a bit unclear if this is the most appropriate system to categorize content from Italy, UK, NZ. Suggest explaining in more detail the rationale behind using this scale.

We thank the reviewer for the comment. The rationale behind using this scale is motivated by the fact that MBFC is one of the most used fact checking authorities in scientific research when it comes to providing a categorization of news outlets. According to this, we provide a list of influential papers that used MBFC to perform the categorization of news outlets:

- Kiran Garimella, Gianmarco De Francisci Morales, Aristides Gionis, and Michael Mathioudakis. 2018. Political Discourse on Social Media: Echo Chambers, Gatekeepers, and the Price of Bipartisanship. In Proceedings of the 2018 World Wide Web Conference (WWW '18).
- Bovet, Alexandre, and Hernán A. Makse. "Influence of fake news in Twitter during the 2016 US presidential election." Nature communications 10.1 (2019): 1-14.

5.) Can engagement with Facebook posts also be assessed in the regression analysis? If the goal is to characterize the relationship between information consumption and epidemiological situation/pandemic media

coverage, I would think that data about engagement should be included in the model as opposed to just looking at the number of posts?

We thank the reviewer for the comment. Unfortunately, CrowdTangle does not provide the evolution of the engagement of a post. Indeed, the counters referring to the different kinds of engagements (likes, comments, shares, reactions) depend on the time the collection of data happened. Therefore, we cannot include this feature in the regression analysis since we do not have a proper time framing. Moreover, the regression analysis would not benefit from engagement since we should also take into account the fact that some posts may go viral, resulting in a long-tailed distribution that will not improve the characterization of the potential infodemic on Facebook.

6.) I suggest reconsidering making conclusive claims about "infodemic volume" or "trend of the infodemic" because this study only looks at one specific aspect of the infodemic - I don't think we can assume that data from Facebook and ONCI can be used as proxy measures for broader information environment, which includes other social media platforms, closed online groups, offline conversations, etc. (also, again, data from CrowdTangle is restricted to public content and excludes content generated by "regular" consumers").

We thank the reviewer for the comment. We rephrased some sentences concerning the terms stated by the reviewer in order to make more precise claims. Specifically, in Section 3, Page 7-8, we estimate the *“potential volume of the infodemic”*, the fact that infodemic evolution *“may increase”*, the *“potential increase in the volume of the infodemic”*, the fact that infodemic *“may have a significant relationship with both socio-epidemiological factors”*. As also mentioned in Section 3, the extension of our work to different environments with richer data could provide more details about the interplay between infodemic and pandemic and shed light on the representativeness of CrowdTangle data.
